# Clomipramine potentially induced fatal torsades de pointes in a patient with acute decompensated heart failure: a case report

**DOI:** 10.3389/fpsyt.2025.1595211

**Published:** 2025-07-15

**Authors:** Rong-Hua Wang, Yu-Liang Lu, Jia-Hui Lu

**Affiliations:** ^1^ Department of Pharmacy, HuZhou Central Hospital, The Fifth School of Clinical Medicine of Zhejiang Chinese Medical University, Huzhou, China; ^2^ Department of Cardiology, HuZhou Central Hospital, The Fifth School of Clinical Medicine of Zhejiang Chinese Medical University, Huzhou, China

**Keywords:** clomipramine, torsades de pointes, acute heart failure, QT prolongation, hypokalemia

## Abstract

**Background:**

While clomipramine is generally considered lower risk for QT prolongation among tricyclic antidepressants, its potential to induce torsades de pointes (TdP) remains poorly characterized, particularly in patients with multiple risk factors.

**Case presentation:**

A 78-year-old male with a history of hypertension, atrial fibrillation, and post-stroke depression presented to the emergency department with a one-week history of chest distress. Initial evaluation revealed atrial fibrillation with a prolonged QTc interval of 550 ms on electrocardiogram (ECG) monitoring, and elevated B-type natriuretic peptide (1130 pg/mL). The patient was admitted for acute decompensated heart failure and treated with torasemide intravenously while continuing clomipramine (25 mg daily) for depression. Within 24 hours, he experienced multiple episodes of torsades de pointes (TdP), coinciding with hypokalemia (serum potassium: 3.21 mmol/L). Despite corrective measures, including potassium and magnesium supplementation, the patient developed ventricular fibrillation and cardiac arrest, leading to death.

**Conclusion:**

This case highlights the potential risk of clomipramine-induced QT prolongation and TdP, particularly in patients with acute heart failure and electrolyte imbalances, underscoring the need for careful risk assessment and monitoring in such populations.

## Introduction

The cardiac safety profile of antipsychotics and antidepressants has been of clinical concern for decades (1). A number of antidepressants are assumed to increase the risk of malignant arrhythmias and cardiac death primarily due to the potential to prolong QT intervals (2). Nevertheless, clomipramine, a tricyclic antidepressant, is considered to have a relatively lower risk of QT prolongation compared to other agents in its class (3). Furthermore, it has not been explicitly associated with torsades de pointes (TdP), a potentially fatal ventricular tachycardia. To date, information about clomipramine related TdP has been limited to sporadic cases documented in the World Health adverse reporting data base(vigibase) (4). Herein, we present a case report further to explore the potential causal relationship between clomipramine and TdP, aiming to enhance understanding of the risks associated with clomipramine use in specific clinical scenarios.

## Case presentation

A 78-year-old man presented to the emergency department complaining of chest distress, appetite loss, and decreased urine output over the course of a week. His medical history included hypertension for approximately ten years and atrial fibrillation for four years. Three years prior, he experienced a cerebral infarction, subsequently developing post-stroke depression syndrome.

Upon admission, the patient’s vital signs were recorded: body temperature, 37.7°C; blood pressure, 150/97 mmHg; and pulse rate, 83 beats/minute. Physical examination of the precordial region revealed no abnormalities, with a heart rate of 93 beats/minute, an irregular rhythm, and no audible murmurs across the valve areas. Lung auscultation identified bilaterally coarse breath sounds and scattered wet rales in the lower lung fields. Mild edema was observed in both lower extremities. The neurological assessment demonstrated a muscle strength grade of IV in the right limb and grade III in the left limb.

Initial laboratory findings were unremarkable for routine blood chemistry and liver function tests, with note of a serum potassium level of 4.32 mmol/L and a creatinine level of 106.3 μmol/L. Notably, the estimated creatinine clearance, calculated by the Modification of Diet in Renal Disease (MDRD) equation, was 62 mL/min/1.73 m². Cardiac biomarkers revealed a markedly elevated B-type natriuretic peptide (BNP) level of 1130 pg/mL and a borderline troponin I level of 0.036 ng/mL (cut-off <0.04 ng/mL). Computed tomography (CT) imaging showed scattered exudative foci in both lungs and bilateral pleural effusion.

**Figure 1:**
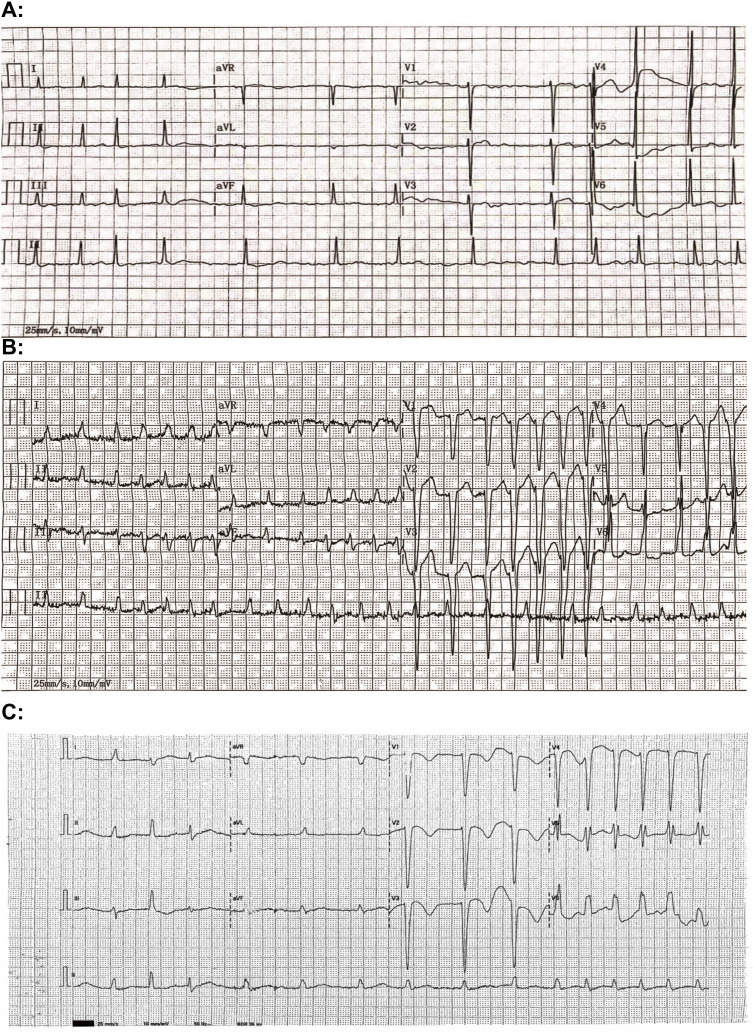
Electrocardiogram (ECG) tracings from 4 September 2020 **(A)**, 16 December 2023 **(B)**, and 26 December 2023 **(C)** reveal the corrected QT (QTc) intervals of 426 milliseconds (ms), 508 ms, and 550 ms, respectively.

An electrocardiogram (ECG) performed at 22:16 upon admission revealed atrial fibrillation with left bundle branch block and non-sustained ventricular tachycardia. The corrected QT interval (QTc), calculated manually using the Bazett formula, was prolonged at 550 milliseconds (ms) (**Figure 1**).

The patient was admitted to the cardiovascular ward with a provisional diagnosis of acute heart failure. The patient’s medication history was collected, including clomipramine (25 mg once daily) for depression, warfarin (1.25 mg once daily) for anticoagulation therapy, and clonazepam (1 mg once daily) for sleep disorder. No rhythm control medication for atrial fibrillation was reported. Initial treatment consisted of intravenous torasemide (20 mg, stat) combined with oral spironolactone (20 mg once daily) for symptomatic diuretic treatment and subcutaneous enoxaparin (40 mg) for anticoagulation. At 22:45, a maintenance dose of 25 mg clomipramine was administered orally to continue the ongoing therapy.

**Figure 2:**
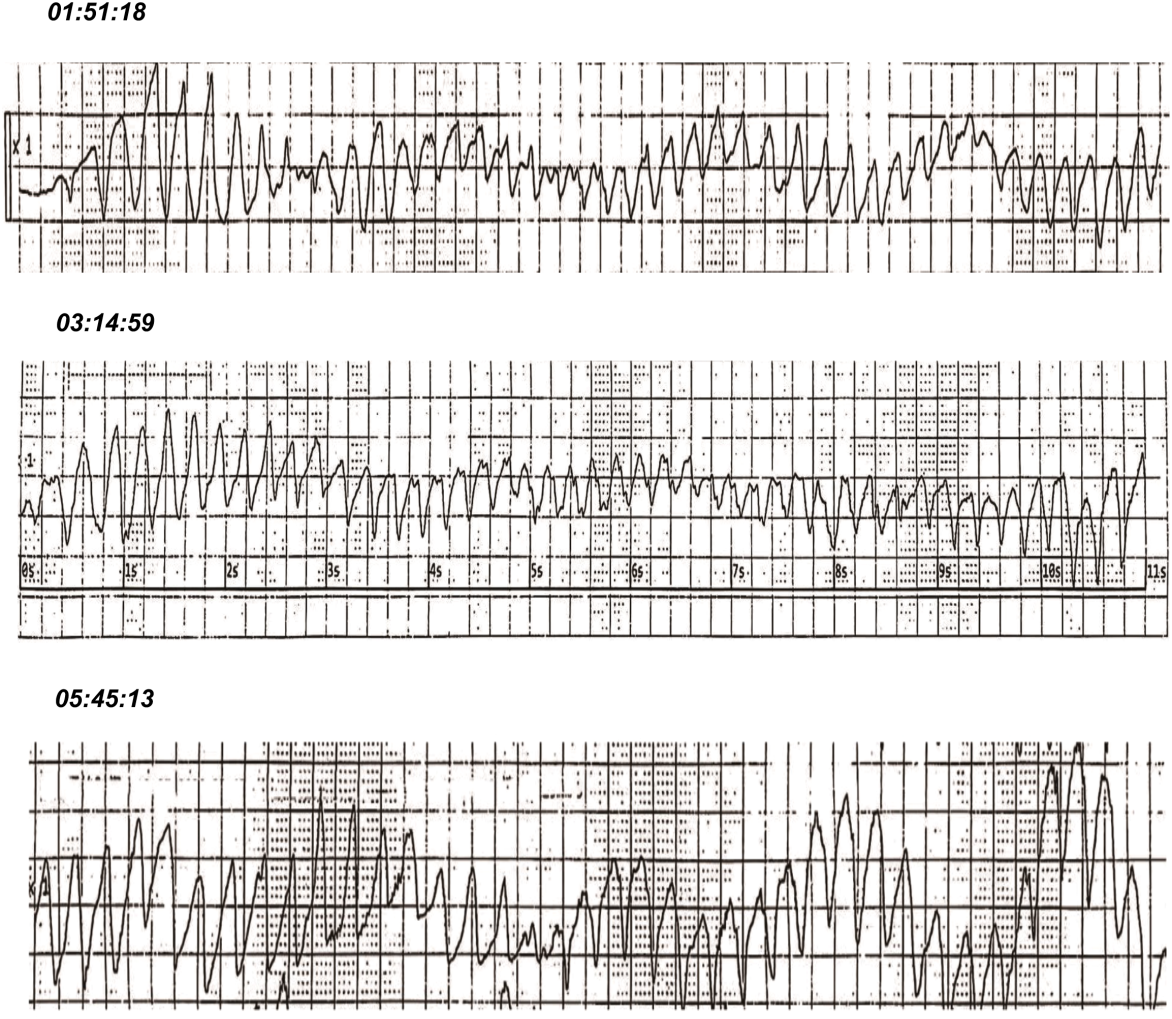
Electrocardiographic (ECG) recordings captured recurrent episodes of torsades de pointes (TdP) approximately 3 hours after administration of a 25 mg maintenance dose of clomipramine upon hospital admission.

Continuous ECG monitoring for the patient indicated underlying atrial fibrillation with frequent episodes of ventricular tachycardia. The initial episode of torsades de pointes (TdP) was noted at 01:51 a.m. on the following day (**Figure 2**), accompanied by the patient’s report of chest tightness and palpitations. A dose of 0.2mg digoxin injection was given intravenously to control the heart rate. Over the next four hours, the patient experienced two additional episodes of TdP, each lasting less than 20 seconds and resolving spontaneously. An emergent chemistry panel revealed a decreased serum potassium level of 3.21 mmol/L. In response, magnesium and potassium supplements were promptly administered, and esmolol was prescribed to mitigate the prolonged QT duration. Fluid balance monitoring revealed a net negative balance of 500 mL during hospitalization. However, these interventions demonstrated limited efficacy, as frequent episodes of ventricular tachycardia persisted. The patient’s condition was deemed critical, with a high risk of progression to ventricular fibrillation, cardiac arrest, or respiratory arrest at any time.

At approximately 1:00 p.m. on the second day of hospitalization, the patient suddenly lost consciousness, became unresponsive to stimuli, and exhibited no palpable pulse in major arteries. ECG monitoring confirmed ventricular fibrillation. Immediate resuscitation measures, including chest compressions, electrical defibrillation, and intravenous administration of 1 mg epinephrine, were performed. Subsequent ECG monitoring detected an escape rhythm, but blood pressure and oxygen saturation remained undetectable. At 1:34 p.m., the ECG displayed asystole, confirming the cessation of breathing and pulse. The patient was pronounced deceased at this time.

## Discussion

Most tricyclic antidepressants (TCAs) are acknowledged as having a propensity to prolong the QT interval, although documented cases of torsades de pointes (TdP) remain relatively rare and are primarily associated with amitriptyline and maprotiline (1). Our case report characterized in detail a patient receiving a therapeutic dose of clomipramine who developed QT interval prolongation and subsequent recurrent episodes of TdP during hospitalization for acute decompensated heart failure. Furthermore, hypokalemia secondary to diuretic use, along with the clinical condition of heart failure, were recognized as synergistic risk factors contributing to the fatal arrhythmic event potentially induced by clomipramine.

The potential of clomipramine to prolong the QT interval has yielded inconsistent findings across studies. Some researchers have reported that the risk of QT prolongation associated with clomipramine is not clinically significant, as demonstrated in a prospective cohort study (5). On the contrary, an *in vitro* study has revealed that clomipramine induces a concentration-dependent blockade of the hERG (human ether-à-go-go-related gene)-encoded potassium channel, establishing an electrophysiological mechanism for QT interval prolongation and increased susceptibility to TdP (6). Accordingly, the CredibleMeds website, a widely recognized academic resource for evaluating drug associated TdP risk, categorizes clomipramine as a “conditional risk” medication, reflecting its relatively lower risk stratification for TdP (7).

**Figure 3:**
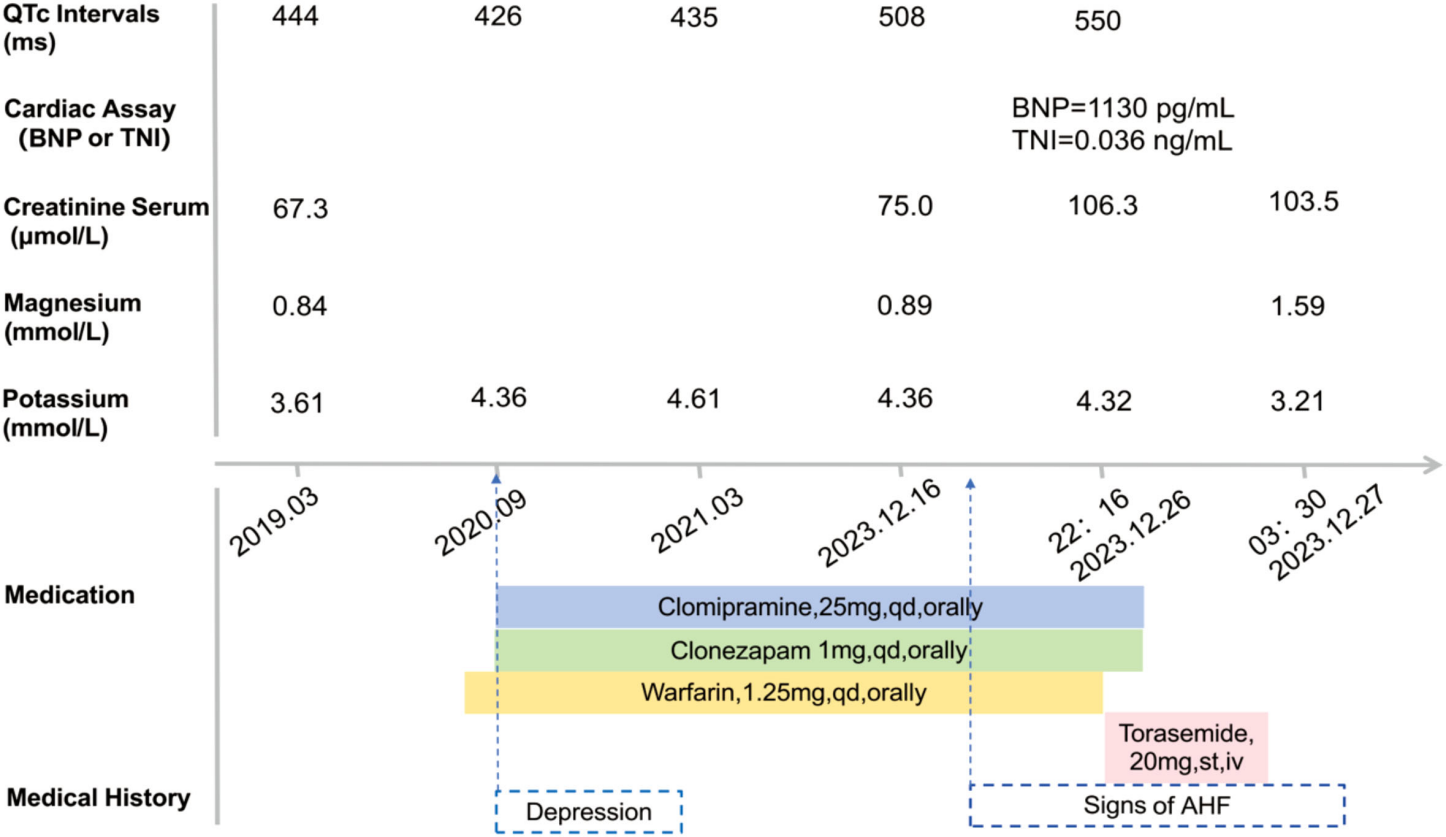
Patient case timeline: laboratory assay data and QTc Intervals on electrocardiogram (ECG) record. QTc, corrected QT interval (milliseconds, ms); BNP, B-type natriuretic peptide; TNI, troponin I; AHF, Acute Heart Failure.

For this patient, a comprehensive medication history was meticulously reviewed, and relevant clinical findings were retrospectively analyzed in a timeline (**Figure 3**). Annual serial ECG monitoring demonstrated a normal baseline QT interval prior to the treatment with clomipramine (**Figure 3**), ruling out congenital long QT syndrome. Following the initiation of low-dose clomipramine (25 mg daily) on September 4, 2020, subsequent ECG monitoring on March 8, 2021, revealed a preserved QT interval of 435 ms. Notably, during a routine follow-up ten days before his last hospitalization, the patient remained free of clinical signs of heart failure. However, the ECG showed clinically significant QTc prolongation to 508 ms (manually measured and corrected by the Bazett Formula, **Figure 1B**), meeting the predefined criteria in the thorough QT study (TQT) (8). Normal serum levels of potassium (4.32 mmol/L) and magnesium (0.85 mmol/L) suggested that a drug-induced effect probably contributed to the observed QTc interval prolongation (9). The extended use and cumulative concentration of clomipramine were presumed to be responsible for QTc interval progressive prolongation, as none of the concurrently prescribed medications, including warfarin and clonazepam, are associated with TdP risk according to the CredibleMeds database (7). During hospitalization, the maintenance of clomipramine combined with a vulnerable condition, including hypokalemia, QTc intervals prolongation to 550ms, and heart failure, constituted the precipitating factor for the fatal arrhythmic event.

Several individual risk factors for developing TdP could be identified in this case involving clomipramine. First, the acute decompensated heart failure condition probably predisposed the patient to an altered pharmacokinetic profile for clomipramine. Previous studies have indicated that plasma concentrations of drugs may increase up to 1.5-fold or more from baseline in the setting of declining hepatic or renal function secondary to acute heart failure (10). Concurrent with the decreased urine output as a manifestation of acute heart failure prior to the emergency visit, serum creatinine level markedly rose by 30 μmol/L from the last follow-up baseline of 75 μmol/L to 106.5 μmol/L upon admission in this case (11) (**Figure 3**). It implies that impaired renal clearance consequent to acute heart failure likely led to the accumulation of demethylclomipramine, an active metabolite of clomipramine that undergoes primarily renal excretion (12), resulting in plasma concentrations exceeding the threshold for QT prolongation. However, the interpretation remains unverified due to the absence of laboratory data. Additionally, heart failure is a well-established intrinsic factor that enhances sensitivity to drug-induced QT interval prolongation and increases the risk of TdP by altering cardiac conduction pathways (13). Furthermore, a notable decline in serum potassium level from 4.32 mmol/L to 3.21 mmol/L (**Figure 3**) due to decongestive therapy with loop diuretics likely contributed to the exacerbation of QT interval prolongation. Hypokalemia is strongly related to QT interval prolongation and TdP risk through its dual action of inhibiting the IKr potassium channel and potentiating late inward currents, resulting in delayed repolarization that facilitates malignant arrhythmias (13, 14). The convergence of multiple risk factors critically compromised cardiac repolarization reserve, ultimately triggering the observed arrhythmic event. To quantify the individual risk of QT prolongation, we applied a validated risk prediction algorithm with a maximum score of 21 (15). In this case, the calculated score of 12 indicated a high risk of QT interval prolongation (**Table 1**).

**Table 1:** Tisdale risk score assessment for the patient.

Risk Factors	Score	Personal Condition
Age ≥ 68 yrs	1	1
Female sex	1	–
Loop diuretic	1	1
Serum K+ ≤ 3.5 mEq/L	2	2
Admission QTc ≥ 450 ms	2	2
Acute myocardial infarction	2	–
≥ 2 QTc-prolonging drugs	3	–
Sepsis	3	–
Heart failure	3	3
One QTc-prolonging drug	3	3
Maximum/Total Risk Score	21	12

Risk Stratification:

• Low risk: Score ≤ 6

• Moderate risk: Score 7–10

• High risk: Score ≥ 11

K^+^=potassium; mEq/L=milliequivalents per liter; ms=milliseconds.

Several limitations in this case should be acknowledged. The American Heart Association Scientific Statement emphasizes that elevated plasma concentration of QT-prolonging drugs, particularly in the conditions of pharmacokinetic drug-drug interactions or impaired drug elimination, constitutes a pronounced risk factor for TdP (13). While the QT-prolonging effect of tricyclic antidepressants (TCAs) is assumed to be concentrated-dependent (16), we could not obtain the therapeutic drug monitoring (TDM) data of clomipramine and its active metabolite for this patient due to institutional constraints. Moreover, the patient’s genotype for cytochrome P450 (CYP) isoenzyme 2D6 (17), which plays a critical role in clomipramine metabolism, was not tested. This information could have provided further insight into the patient’s potentially poor metabolic status and the resulting aberrant drug concentrations. These knowledge gaps underscore the need for future studies to implement protocolized TDM in high-risk patients receiving QT-prolonging medications, particularly those under compromised drug elimination conditions.

## Conclusion

This case highlights the importance of health providers’ awareness regarding the risk factors associated with QT interval prolongation and torsades de pointes (TdP) in patients prescribed clomipramine. Key risk factors include a history of cardiovascular disease, advanced age, female sex, hypokalemia, impaired elimination potential for rising plasma concentration, and concomitant use of QT-prolonging medications. Prior to initiating clomipramine, a thorough assessment of the patient’s risk for QT interval prolongation is essential. For patients on long-term clomipramine therapy, therapeutic drug monitoring (TDM) of plasma concentrations is recommended to mitigate the risk of drug accumulation and subsequent toxicity. Clinicians should be well-versed in these risk factors, adept at identifying patients at high risk for clomipramine-induced arrhythmias, and knowledgeable about structurally related medications that may pose similar risks.

## Data Availability

The datasets presented in this article are not readily available because of ethical and privacy restrictions. Requests to access the datasets should be directed to the corresponding authors.
